# Reversible alterations of brain acetate metabolism associated with alcohol consumption

**DOI:** 10.1038/s41386-026-02455-6

**Published:** 2026-05-30

**Authors:** Chathura Kumaragamage, Lihong Jiang, Gustavo A. Angarita, Robin A. de Graaf, Elizabeth Guidone, Anastasia Coppoli, Kevin L. Behar, Barbara I. Gulanski, Brian Pittman, Stuart A. Weinzimer, Douglas L. Rothman, John H. Krystal, Graeme F. Mason

**Affiliations:** 1 Department of Radiology, Cumming School of Medicine, University of Calgary, Calgary, AB Canada; 2 Department of Radiology and Biomedical Imaging, Yale Biomedical Imaging Institute, Magnetic Resonance Research Center, New Haven, CT USA; 3 Department of Psychiatry, Yale University, New Haven, CT USA; 4 Department of Biomedical Engineering, Yale University, New Haven, CT USA; 5 Department of Internal Medicine, Yale University, New Haven, CT USA

**Keywords:** Mechanisms of disease, Cellular neuroscience, Neurochemistry

## Abstract

Alcohol consumption elevates circulating acetate. Prior studies showed that acute alcohol reduces brain glucose uptake and increases brain acetate oxidation. Previously we showed that heavy drinkers have elevated capacity to oxidize brain acetate. Here we repeat the study, adding individuals with alcohol use disorder (AUD). Four groups were enrolled. The analysis data set included Light Drinkers (LD, *n* = 13, female = 5), at-risk Heavy Drinkers (HD, *n* = 15, female = 7), AUD patients in long-term recovery (≥6 months; AUD_LTR_, *n* = 6, female = 1), and a separate group of AUD treatment-seekers (AUD_Tx_, *n* = 12, female = 1) underwent medically supervised detoxification, scanned at ~1 week abstinence (*n* = 9) and 1 month (*n* = 10). Seven AUD_Tx_ participants successfully completed scans at both time points. We infused participants with [2-^13^C]acetate during magnetic resonance spectroscopy (MRS) of ^13^C-glutamate (Glu) and ^13^C-glutamine (Gln) in the brain, to measure the cerebral metabolic rate for acetate (CMR_Ac_) and the neuronal tricarboxylic acid cycle relative to glutamate-glutamine neurotransmitter cycling (V_tcaN_/V_cycle_; Energy Per Cycle: EPC). There was a group effect for CMR_Ac_ (*p* = 0.007) primarily owing to lower CMR_Ac_ in AUD_Tx_ at 1 week. Furthermore, higher CMR_Ac_ was observed among HD compared to LD participants, as previously reported. CMR_Ac_ was similar between the AUD_LTR_ and HD groups. In a separate within-subject comparison among AUD_Tx_ participants, CMR_Ac_ increased after 1 month to levels similar to those of LD. EPC was similar among the groups, representing normal glutamate-glutamine cycling versus energetics. In summary, abstinence reversed the lower acetate oxidation in early AUD, showing that just a few weeks of recovery can normalize this metabolic abnormality.

## Introduction

AUD refers to a problematic pattern of alcohol consumption that can lead to significant harm or distress. It ranges from mild maladaptive drinking to severe dependence [1]. Symptoms include excessive drinking, loss of control over alcohol intake, withdrawal symptoms, tolerance, and continued use despite negative consequences [2]. Heavy alcohol consumption increases risks of many adverse health consequences, including liver disease and cancer [3–5]. Approximately 95,000 alcohol-related deaths occur annually, making it the third highest preventable cause of death in the USA [6]. The economic impact of AUD is significant and widespread [7].

Acute alcohol intoxication shifts the brain’s excitation/inhibitory balance toward inhibition, via events that include enhanced γ-aminobutyric acid (GABA)-mediated inhibition and reduced glutamatergic excitatory tone [8]. Chronic drinking leads to compensatory adaptations of increased excitatory glutamatergic capacity and reduced GABA-mediated inhibition to counteract the prolonged excitation/inhibition imbalance. These mechanisms are central to the development of alcohol withdrawal symptoms [1, 9, 10].

A standard treatment to manage alcohol withdrawal includes benzodiazepines, which enhance GABAergic inhibition via the GABA_A_ receptor [10]. However, the potential for abuse, dependence, or overdose of benzodiazepines requires close monitoring, especially when combined with alcohol and other medications [11, 12]. Positive and negative reinforcement mechanisms of alcohol withdrawal are hypothesized to promote alcohol consumption and subsequent relapse [13]. As such, advancing our understanding of underlying neurobiological consequences of chronic alcohol use is critical to develop effective therapies for AUD that can minimize withdrawal symptoms and promote sobriety [1].

Oxidative glucose metabolism is the primary energy source in the human brain, but acetate, which is exclusively utilized by astroglia, can provide an alternate substrate for those cells [14]. Acute alcohol consumption increases acetate production in hepatic mitochondria and raises blood acetate levels [15], which increases brain acetate metabolism and reduces glucose metabolism [16–19]. While acute ethanol intoxication alters glucose metabolism, habitual risky ethanol consumption can enhance acetate oxidation in the brain [20]. The effect is greater in heavy drinkers [17] and persists beyond the state of intoxication [17, 21]. Alcohol’s reinforcing effects may result not only from enhancement of pharmacologic action on neurotransmitters [22–24], but also from energetic changes.

Alcohol metabolism consists of conversion to acetaldehyde by alcohol dehydrogenase, catalase, or cytochrome P450, then subsequent modification to acetate by aldehyde dehydrogenase. Acetaldehyde is highly toxic and carcinogenic, so the conversion of acetaldehyde to acetate is very efficient. Acetate then serves as an energy source, substrate for biosynthesis, and signal regulator of other pathways [25–28]. As a fuel, acetate enters the tricarboxylic acid (TCA) cycle to provide ATP for cerebral and systemic energy needs. Acetate also plays an important role in fatty acid metabolism and protein modifications that affect metabolic regulation and gene expression [29]. In brain, acetate is selectively utilized by astrocytes [30], so isotopically labeled acetate can be used to trace glial metabolism and the glutamate-glutamine neurotransmitter cycle [31–33], which relates directly to the rate of synaptic glutamate release.

^13^C MRS with ^13^C-acetate administration allows assessments of neuroenergetics in vivo, including the capacity for acetate consumption and the rates of the neuronal tricarboxylic acid cycle (V_tcaN_) relative to glutamate-glutamine neurotransmitter cycling (V_cycle_) [32–38]. Determination of V_tcaN_/V_cycle_ is of interest because it represents neuronal energy consumption relative to glutamate release. The relationship provides a measure of Energy Per Cycle (EPC), proposed as a marker of glutamatergic synaptic strength [38]. The value of the ratio V_tcaN_/V_cycle_ is preserved across many species [39–41], laboratories, and brain regions [42], but it is altered in some conditions [38, 43] and has potential as a target for novel therapies.

Previously we showed that at-risk HD have upregulated brain acetate oxidation [17]. The HD group and LD groups in that pilot study were small (*n* = 8 for each). In this study, we recruited new, larger LD and HD groups. We also recruited two new groups: individuals in recovery longer than 6 months (AUD_LTR_) and treatment-seeking subjects with AUD going through detoxification (AUD_Tx_). We tested for impacts of chronic drinking and abstinence on brain acetate oxidation in all groups. We hypothesized that we would reproduce our original finding that HD subjects had higher brain consumption of acetate relative to LD subjects. For the AUD group, we hypothesized that brain uptake and consumption of acetate would be far more than the LD or HD groups, and that over time there would be a return to normalcy in the AUD_LTR_ group. We also examined EPC across groups.

## Material and methods

Screening and testing protocols were approved by the Yale University Institutional Human Investigation Committee and Yale University Magnetic Resonance Research Center (MRRC) Protocol Review Committee. Written informed consent was obtained from all participants after a complete explanation of the study procedure.

### Participants

Four groups were included in the study: Light/Non-Drinkers (LD, *n* = 15, 6 women), at-risk Heavy Drinkers (HD, *n* = 15, 7 women), individuals with AUD undergoing medically supervised inpatient detoxification at ~1 week abstinence (AUD_Tx_(1 W), *n* = 14), and individuals in long-term recovery (≥6 months; AUD_LTR_, *n* = 6). Recruitment was via flyers and advertisements posted in the New Haven area. Eligibility for the LD group required that participants consume fewer than 3 standard drinks per week. The HD group was defined as men who consume more than 14, or women who consume more than 11 standard drinks per week over the past 6 months, similar to our previous study [17]. Of the 12 AUD_Tx_(1W) subjects, 11 completed treatment and enrolled in the second session after ~1 month of abstinence, forming the AUD_Tx_(1M) group. The AUD_LTR_ group was recruited by flyers and word of mouth, except for three subjects who participated in the AUD_Tx_ group and stayed in contact with us. Abstinence was assessed by a breathalyzer reading of 0, and we verified normal liver function test results. For the three subjects that also participated in the AUD_Tx_(1W) and AUD_Tx_(1M) groups, a family member or confidant verified abstinence for each subject periodically before the AUD_LTR_ scan.

Of those who completed scans, some subjects were excluded: it was not possible to measure plasma acetate concentrations in two LD and three AUD_Tx_(1W) subjects due to problems with blood sampling, two of which also had uninterpretable data due to motion. One AUD_Tx_(1M) subject was excluded because plasma acetate and brain labeling never reached a steady state, which precluded reliable kinetic analyses and comparison of steady-state labeling with the other subjects. Another AUD_Tx_ participant self-discharged before the one-month scan. Each group yielded usable data as follows: 13 LD, 15 HD, 9 AUD_Tx_(1W), 10 AUD_Tx_(1M), and 6 AUD_LTR_.

Detailed inclusion and exclusion criteria are provided in the Supplemental Material. Participants who met inclusion criteria based on a phone screen had routine laboratory tests, including CBC, glucose, BUN/Creatinine, electrolytes, folate, B_12_, liver and thyroid function tests, urine drug screen, and urine pregnancy test for women of child-bearing potential. A psychiatric screening was completed using the structured Clinical Interview for DSM-IV Axis I Disorders (SCID I). General demographics were obtained, including alcohol and tobacco consumption over the 60 days before the testing, by the Timeline Follow Back method (TLFB) [44]. Exclusion criteria included history of neurological disorders, contraindications to magnetic resonance measurements, and substance dependence history other than alcohol (for LD and HD groups) or tobacco. Subjects were instructed to abstain from alcohol 24 h before the study and to fast after 10 pm the evening before. Smokers were allowed to use nicotine until one hour before the scan. Supplementary Table S1 summarizes the demographics of all participants. 10/14 subjects in the AUD_Tx_(1W) group required benzodiazepines to stabilize withdrawal symptoms.

### Subject preparation and acetate administration

On the study day, subjects reported to the MRRC at 8:00am, underwent a brief physical examination and placement of antecubital intravenous (IV) lines in each arm (one to infuse acetate, the other to draw blood). The Investigational Drug Service at Yale New Haven Hospital Pharmacy prepared sodium [2-^13^C]acetate as a 350 mM solution in water. The acetate infusion protocol consisted of a 5-min bolus at a rate of 6 mg/kg/min, followed by a steady infusion rate of 3 mg/kg/min to complete the 2-h infusion period [45].

### Magnetic resonance spectroscopy (MRS)

Localized ^13^C-MRS was performed in vivo using a Bruker (Bruker BioSpin, Billerica, MA, USA) imaging spectrometer operating at a magnetic field of 4 Tesla (Oxford Magnet Technology) and running ParaVision 6.0. Subjects lay supine in the magnet, with the head over a radiofrequency magnetic resonance probe consisting of one 8.5-cm diameter circular ^13^C coil and two ^1^H quadrature-driven coils for acquisition and decoupling, respectively [46]. First- and second-order shims were adjusted according to B_0_ field mapping over a cubical voxel measuring 50 × 40 × 45 mm^3^ in the occipital cortex, followed by a localized iterative first-order shim. ^13^C MRS were acquired using polarization transfer optimized to detect glutamate and glutamine carbon 4 (Glu4, Gln4) [46], with ISIS localization [47], a repetition time (TR) of 3.3 s, and 96 averages per block (NA). The free induction acquisition was 102 ms in length, consisting of 512 complex points at a sampling rate of 5000 Hz with WALTZ-16 ^1^H decoupling. The MR spectra were analyzed by a blinded team member (AC) with in-house spectral fitting using a basis set containing acetate C2 (Ace2), Glu4 and Glu3, Gln4 and Gln3, lactate C3, GABA C2 and C3, aspartate C3, and the N-acetylaspartate (NAA) C3 and C6 carbon positions. Because NAA C3 was not enriched noticeably during the acetate infusion, its natural abundance signal was used as a reference to determine the ^13^C-labeled concentrations of Glu4 and Gln4. Subsequent conversion to percent enrichment was done by normalizing to the concentrations of glutamate and glutamine previously determined by ^13^C MRS.

### Plasma acetate measurements

Plasma acetate concentrations were measured by mixing 50 μL of plasma with 50 μL of 10 mM sodium formate, then dissolving in 600 μL of a solution of 40% D_2_O and 60% 50 mM phosphate buffer, pH 7.4. The resulting solution was measured with ^1^H MRS using a 500 MHz Bruker spectrometer running Topspin 3.2, using a pulse-acquire sequence (TR = 25 s, NA = 64). Acetate concentrations were calculated by the relative integrals of the acetate and formate resonances. ^13^C-acetate percent enrichments were determined from the ratios of the ^13^C-coupled satellites relative to their sum with the central uncoupled resonance of acetate (i.e., unlabeled acetate) in the ^1^H spectrum [17].

### Assessment of CMR_ac_/[plasma acetate] by metabolic modeling analysis

Time-resolved ^13^C enriched metabolites labeled during the [2-^13^C]-acetate infusion were used to determine CMR_Ac_ by fitting a 2-compartment model of astroglial and neuronal metabolism using the plasma [2-^13^C]-acetate concentrations and enrichments as inputs [32]. Mass and isotopic fluxes from [2-¹³C]acetate to brain glutamate and glutamine were modeled using CWave 3.2 [17]. CWave is a software package that allows users to define metabolic pools, pathways, and substrates, with concentrations and enrichments as data. CWave generates differential equations and solves them, doing least-square fitting to the data to estimate parameters, in this case CMR_Ac_. The values of CMR_Ac_ were normalized by the steady-state plasma acetate concentration, to account for the dependence of brain CMR_Ac_ on plasma acetate levels.

### Calculation of energy per cycle

The final hour, steady-state enrichments of Glu4 and Gln4 were used to calculate (V_tcaN_/V_cycle_), as described previously [33], according to

$$\frac{{V}_{{tcaN}}}{{V}_{{cycle}}}=\frac{1-\frac{{{PE}}_{{Glu}4}}{{{PE}}_{G{{\mathrm{ln}}}4}}}{\frac{{{PE}}_{{Glu}4}}{{{PE}}_{G{{\mathrm{ln}}}4}}}={EPC}$$

### Statistics

The two primary hypotheses were (1) brain acetate consumption capacity is greater in HD compared to LD subjects, testing reproducibility of our previous report, and (2) brain acetate consumption in AUD_Tx_ differs at 1 week and 1 month (Specific Aims 1 and 2 from the R01 that supported the work). We next wanted to know if AUD_Tx_(1M) and AUD_LTR_ would approach ostensibly normal levels as measured in LD. Each outcome was analyzed with linear mixed models (LMM) with group (between-subjects), time (within-subjects), and the group by time interaction as predictors. Post-hoc pairwise testing assessed steady-state differences among groups. Among AUD_Tx_, time courses for each outcome (measured at ~1 week and 1 month) were compared using LMM with session and time both modeled as within-subjects factors. Separate post-hoc analyses compared acetate levels between AUD_Tx_ measured at 1 month to LD to determine the extent of normalization after abstinence. False discovery rate (FDR) corrections were applied for post hoc comparisons involving more than two comparisons [48], with raw and corrected p-values reported.

## Results

### Plasma acetate

**Fig. 1:**
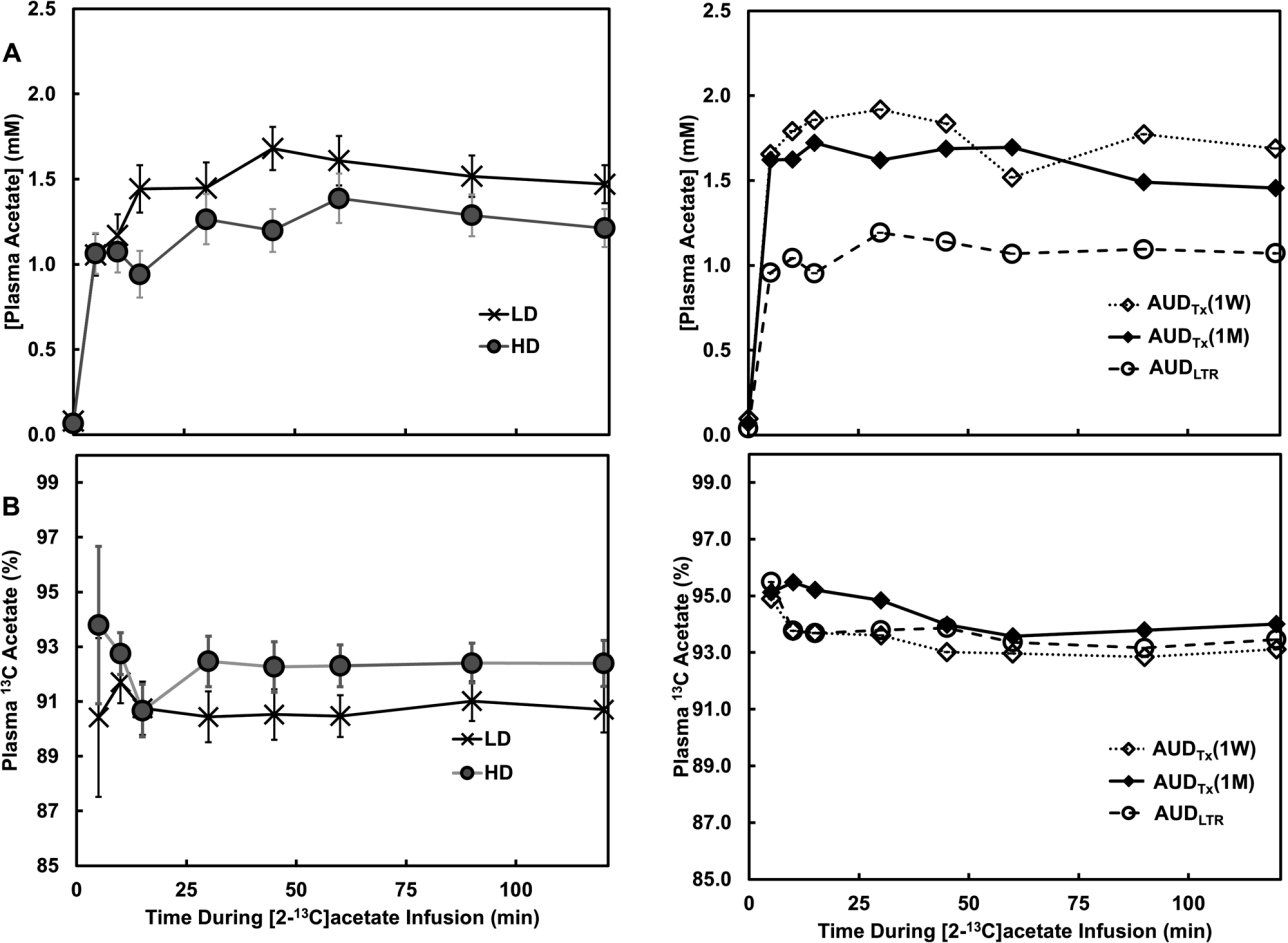
Plasma acetate concentrations and ^13^C-enrichments during infusion of [2-^13^C]acetate using the same acetate infusion protocol: 5-minute bolus of 6 mg/kg/min followed by steady infusion rate of 3 mg/kg/min. **A** Time courses of plasma acetate concentrations. **B** Time courses of plasma ^13^C-acetate enrichments.

During the infusion, plasma acetate concentrations and ^13^C enrichments rose from ~0.1 mM to 1–1.5 mM and 85–95%, respectively, within 5 min (Fig. 1). For concentration, a time effect (*p* < 0.0001) driven by the rapid rise in concentration, and a group effect (*p* = 0.010) driven mainly by higher levels in AUD_Tx_(1W) was found. These effects differed by group (*p*_interaction_ = 0.006). Specifically, post-hoc comparisons showed concentrations in AUD_Tx_(1W) (1.57 ± 0.13 mM) to be higher than HD (1.06 ± 0.10; *p* = 0.0034, *p*_FDR_ = 0.014) and AUD_LTR_ (0.95 ± 0.16; *p* = 0.005, p_FDR_ = 0.014). For enrichments, the group x time interaction (*p* = 0.16) and group effect (*p* = 0.19) were not significant.

Within-subject comparisons of concentrations measured across time for each session (AUD_Tx_1W vs. AUD_Tx_1M) showed similar plasma acetate levels at 1 week and 1 month (*p*_session_ = 0.5). While the overall pattern of acetate time courses differed between 1 week and 1 month (*p*_interaction_ = 0.046), levels were similar at each time point between sessions. In terms of enrichment, while there was no group x time interaction observed (*p* = 0.90), overall levels were greater when measured at 1 month compared to 1 week (*p*_session_ = 0.039).

Considering normalization of metabolism with abstinence, we compared LD and AUD_Tx_(1M) groups post-hoc. Concentrations showed no group (*p* = 0.31) or group x time (*p* = 0.45) effects, but there a time-effect (*p* < 0.0001) occurred from the rise from baseline to steady state. Enrichments also showed a group (*p* = 0.049) but no group x time effects (*p* = 0.13). A time effect (*p* < 0.003) was observed that was only 1–2% relative to the enrichment of ~92%.

### ^13^C-labeling of Glu4 and Gln4, normalized by the concentration of plasma ^13^C-acetate

**Fig. 2:**
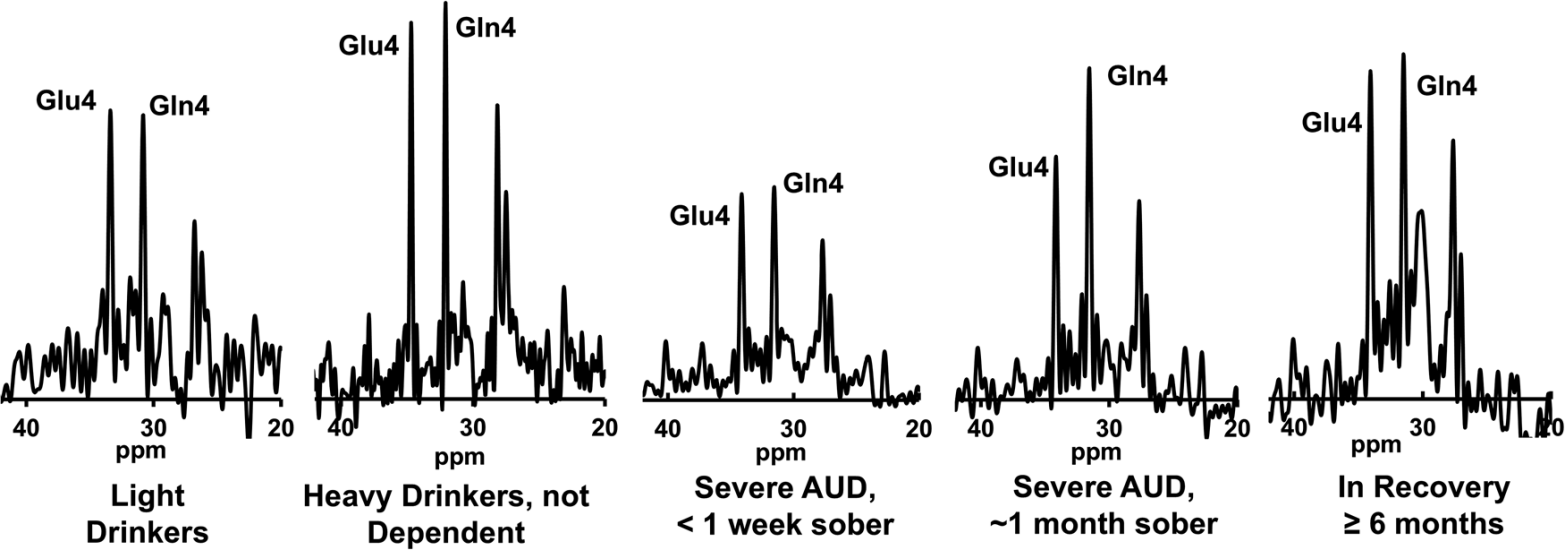
Representative ^13^C MR spectra from each group (LD, HD, AUD_Tx_(1W), AUD_Tx_(1M), and AUD_LTR_), normalized to the concentration of plasma [2-^13^C]acetate, averaged over the final hour of the acetate. Noise levels vary according to the normalization and position of the volume of interest relative to the ^13^C MR surface coil.

**Fig. 3:**
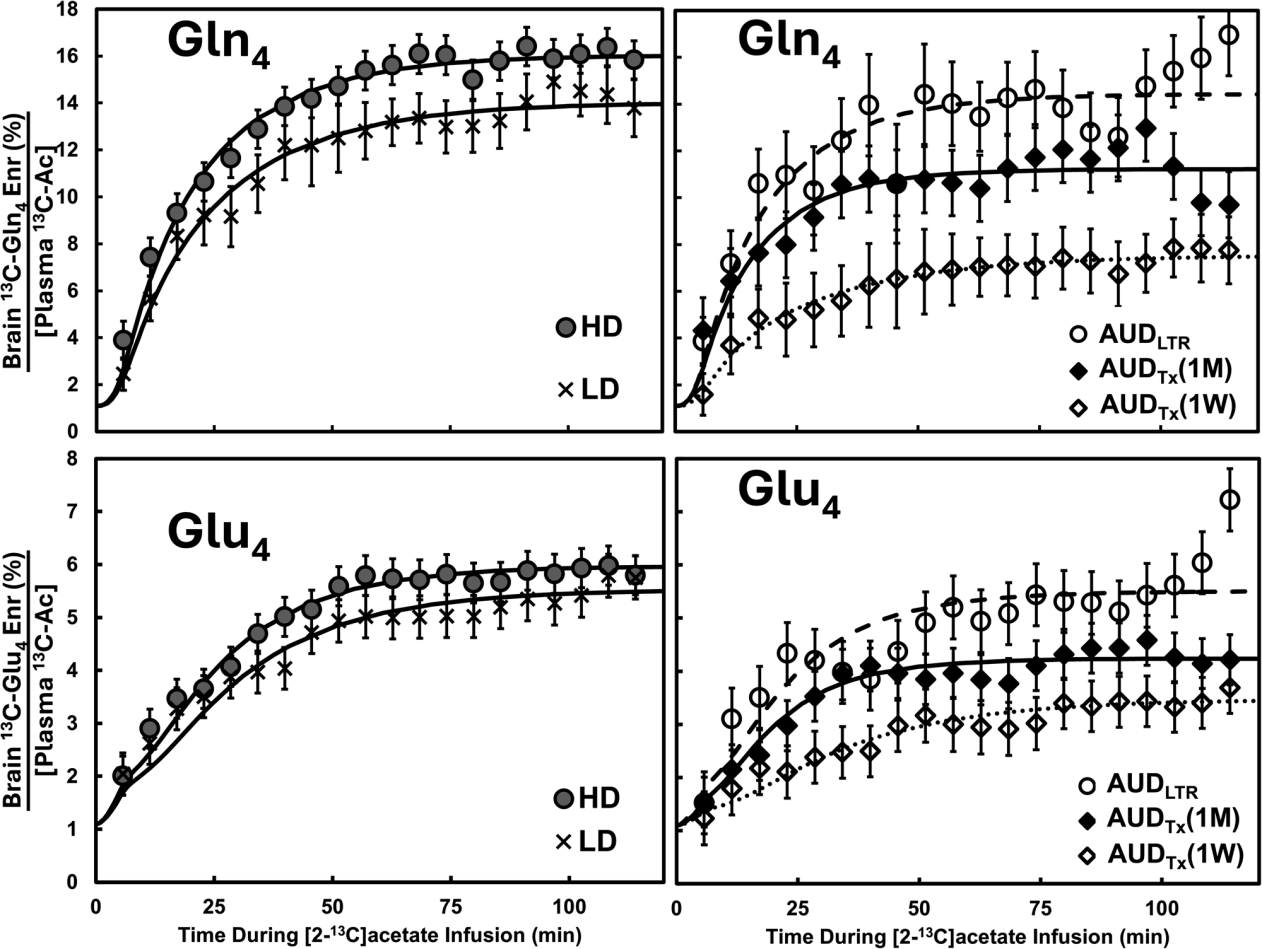
Brain acetate oxidation in each of the groups, leading to ^13^C-labeling of Glu_4_ and Gln_4_, with the fits of the metabolic model as solid curves. The treatment-seeking group showed less ^13^C-labeling in Glu_4_ and Gln_4_ at one week and shifted largely to normal after one month of abstinence, suggesting a state phenomenon.

Figure 2 shows ^13^C brain spectra from individuals in each group. The AUD_LTR_ spectra resemble those of LD and HD. In this range of plasma acetate levels, the conversion of ^13^C-acetate to labeled Gln4 and Glu4 depends linearly on [^13^C-acetate] [32], we normalized Gln4 and Glu4 labeling by the plasma [^13^C-acetate]. Figure 3 shows the average Gln4 and Glu4 time courses normalized to plasma ^13^C-acetate for each group, together with the kinetic fits of the metabolic model. Normalized Gln4 and Glu4 labeling showed group x time effects (*p* = 0.0002, 0.019, respectively), as well as time (*p* < 0.0001 for Gln4 and Glu4) and group (*p* < 0.0001 and *p* = 0.0002). Post hoc, AUD_Tx_(1 W) showed less labeling than LD, HD, and AUD_LTR_ for Gln4 (*p* = 0.0007, <0.0001, and *p* = 0.0009, respectively; *p*_FDR_ = 0.002, *p*_FDR_ = 0.0006; p_FDR_ = 0.002) and for Glu4 (*p* = 0.0008, *p* < 0.0001, and *p* = 0.002, *p*_FDR_ = 0.002, *p*_FDR_ = 0.0006, *p*_FDR_ = 0.004). The AUD_LTR_ group closely resembled the LD and HD groups for Gln4 (*p* = 0.54 for both; *p*_FDR_ = 0.54) and Glu4 (*p* = 0.58 and 0.73; *p*_FDR_ = 0.69 and 0.73).

We previously [17] reported Glu4 and Gln4 labeling from ^13^C-acetate to be higher in HD than LD (30% and 46%, respectively; *p* = 0.01 and p = 0.02). In this new group, the Glu4/Gln4 labeling in HD was insignificantly higher (17% and 11%, respectively; *p* = 0.12 and 0.25, *p*_FDR_ = 0.18 and *p*_FDR_ = 0.37). The smaller difference may be related to less different drinking history between LD and HD: previously, LD and HD groups consumed 1.5 ± 2 and 100 ± 56 drinks in the previous 30 days, respectively, while in the present study, LD and HD groups consumed 2 ± 3 and 76 ± 29 drinks over the previous 30 days.

Within-subject comparisons between AUD_Tx_(1W) and AUD_Tx_(1M) time points showed a session x time effect for Gln4 (*p* = 0.052), a time effect (*p* < 0.0001 for Gln4 and Glu4), and a session effect (0.003 and 0.019, respectively) with Gln4 and Glu4 rising by 1 month to levels approaching the LD group (*p* = 0.37 and 0.11, respectively; Fig. 3).

### Brain acetate oxidation normalized by [plasma ^13^C acetate]

**Fig. 4:**
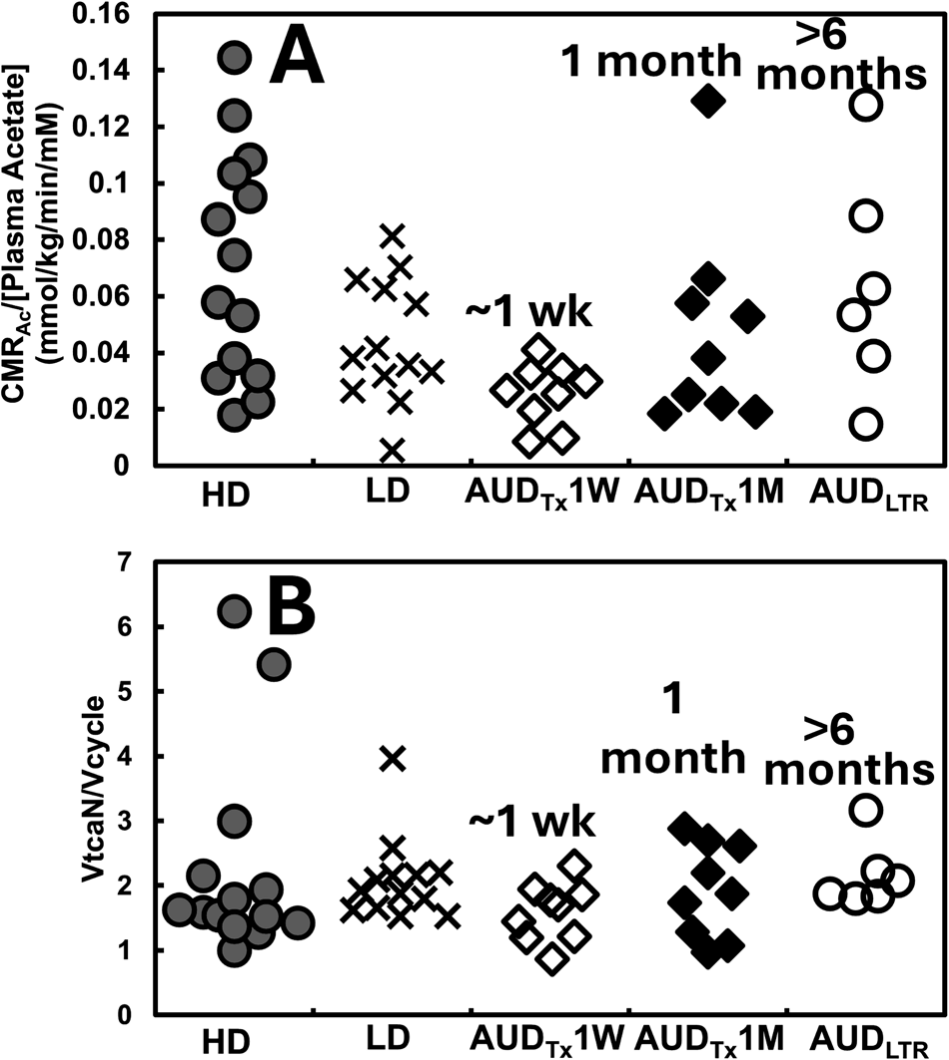
Metabolic rates obtained from ^13^C kinetics during infusions of ^13^C-acetate. **A** Brain acetate oxidation normalized to plasma [2-^13^C]acetate levels from kinetic fitting of the group-averaged time course data. AUD_Tx_(1W) had slower acetate utilization than the other groups, with recovery after one month. Pairwise differences were also observed (LD < HD and AUD_LTR_ and HD < AUD_LTR_). **B** The Energy-Per-Cycle V_tcaN_/V_cycle_ overall showed no significant group differences. The average one-month recovery point AUD_Tx_(1M) appears more similar to LD and AUD_LTR_ for both acetate utilization and EPC.

A significant group effect on normalized CMR_Ac_ was seen (*p*_group_ = 0.007) (Fig. 4A). Post hoc pairwise tests showed lower CMR_Ac_ in AUD_Tx_(1W) compared to HD (64% lower; *p* = 0.0013, *p*_FDR_ = 0.008) and AUD_LTR_ (41% lower; *p* = 0.020, p_FDR_ = 0.06), but not compared to LD (43% lower; *p* = 0.16, p_FDR_ = 0.22). Furthermore, in agreement with our previous work [17], CMR_Ac_ was greater in HD compared to LD (58% higher; *p* = 0.031, p_FDR_ = 0.06).

In a separate within-subject comparison of AUD_Tx_ at 1 week versus AUD_Tx_ at 1 month, CMR_Ac_ increased 43% after 1 month (*p* = 0.053). Furthermore, levels at 1 month normalized when compared to the LD group’s single scan (*p* = 0.77).

### Assessment of V_tcaN_/V_cycle_ (EPC)

There were no differences among groups for EPC (*p* = 0.41).

## Discussion

### Plasma acetate

**Fig. 5:**
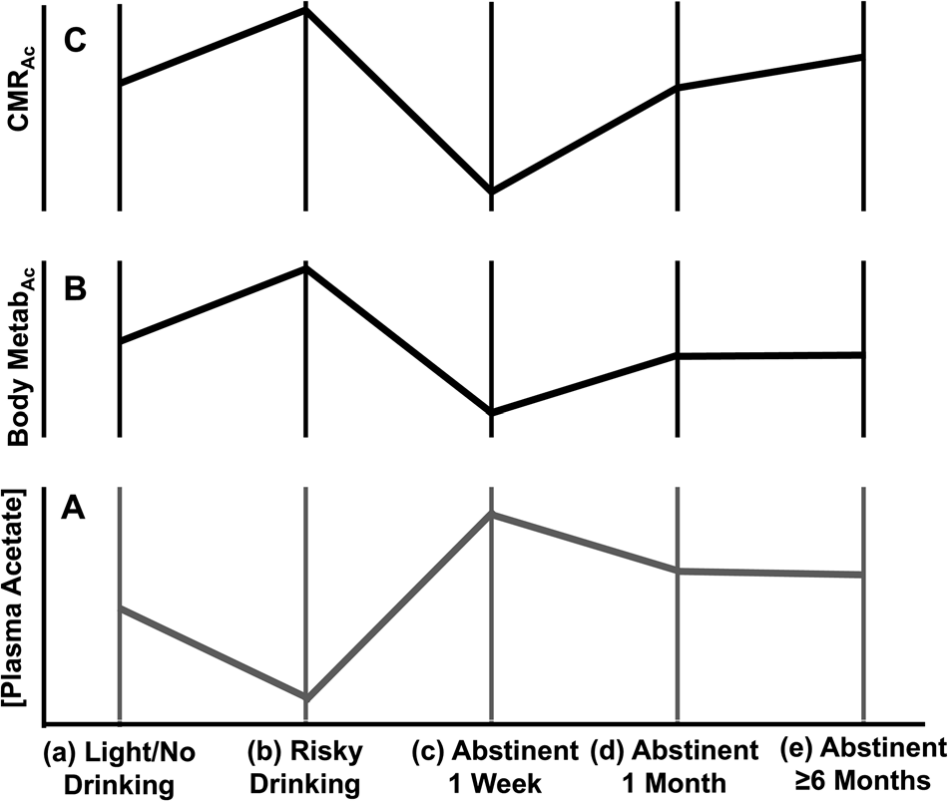
Concept of impact of ethanol on systemic and cerebral metabolism as a progression from social/non-drinking to risky drinking through abstinence and longer-term recovery. Generally, systemic acetate clearance resembles the brain’s capacity to consume acetate. For a fixed dosage of acetate, if plasma acetate levels are lower (**A**), then overall acetate oxidation (Body Metab_Ac_) must be faster (**B**) paralleling cerebral acetate oxidation (CMR_Ac_) (**C**). Compared (a) to light/non-drinking (a), the lower acetate levels with risky drinking (b) require faster systemic acetate consumption, seen also in the brain. Acute abstinence (c) is associated with slower overall acetate consumption, and the brain shows a much slower capacity for acetate consumption. After one month of abstinence (d), the brain acetate oxidation capacity and overall acetate clearance resemble the light/non-drinking condition, appearing to normalize acetate metabolism, with perhaps a small rise after prolonged abstinence (e).

The same infusion protocol was administered for all participants, but higher plasma acetate concentrations were observed in AUD_Tx_(1W) compared to the other groups. Higher plasma acetate levels indicate slower body-wide clearance of acetate. Acetate is consumed readily by skeletal muscle [49–51], heart [52], liver [53], kidney [54], and brown adipose tissue [55]. It appears that shortly after the onset of abstinence, the body clears acetate more slowly. Figure 5 suggests that brain and body acetate consumption parallel one another.

### CMR_Ac_ and ^13^C-labeling of Glu and Gln

The AUD_Tx_ group reported drinking over twice as much alcohol as the HD group, so based on our previous observation of elevated acetate oxidation in HD, we anticipated much greater CMR_Ac_ and brain labeling in AUD_Tx_(1W). However, Glu4 and Gln4 labeling was *lower* in AUD_Tx_(1W), so dramatically that when we scanned our first AUD_Tx_(1W) participant, we initially suspected scanner malfunction. After detoxification (AUD_Tx_(1M)), CMR_Ac_ rose to resemble the LD group. CMR_Ac_ in AUD_LTR_ had an average CMR_Ac_ that resembled CMR_Ac_ in AUD_Tx_(1M), but the small sample and spread of CMR_Ac_ in the AUD_LTR_ group makes it difficult to know if CMR_Ac_ is normal or higher.

Here are potential hypotheses about CMR_Ac_ that are not mutually exclusive:

1. Chronically, plasma acetate (daily, not sporadic) could lead the brain to protect against importing acetate, possibly by reduced MCT across the blood-brain barrier. Mews et al. reported histone acetylation with alcohol exposure in the mouse brain [56], which may be harmful when it occurs chronically [57, 58].
2. Alcohol-induced astrocyte dysfunction [59, 60] could lower acetate oxidation, which could recover with sobriety.
3. Decreased CMR_Ac_ in AUD_Tx_(1W) is unlikely to be related to withdrawal symptoms, as indicated by the low CIWA assessments (Supplementary Table S1). The low CMR_Ac_ in AUD_Tx_(1W) could arise from slower metabolism due to rapid reversal of the hyperexcitable state that follows the removal of alcohol from the system.

Alterations in acetate consumption are not unprecedented: Type 1 diabetes with hypoglycemia unawareness have been shown to be associated with elevated acetate consumption [45, 61]. Over several decades we have noticed that some individuals who are underweight have elevated acetate consumption. Those findings represent cases in which the body relies on non-glucose substrates for energy, as may be the case with chronic, intense, heavy drinking.

### Assessment of EPC

Previous studies have shown reduced EPC in major depressive disorder [37] and post-traumatic stress disorder (PTSD) [43]. A follow up human PTSD study showed a rebound of EPC upon administration of ketamine [38]. Here we investigated EPC across the groups because of hyperexcitability early in detoxification, but we saw no differences. The AUD_Tx_(1W) group was studied after withdrawal symptoms had mostly subsided, so we cannot rule out a difference earlier in detoxification.

### General

Our demographics are consistent with previous reports that the age of the first drink is strongly association with subsequent drinking behavior [62, 63]: LD started drinking at age 20, while HD and AUD began at age 15.

Though addiction-related neurocircuitry primarily involves prefrontal, striatal, and limbic regions, the occipital cortex was used here for methodological and biological reasons. The occipital lobe is the largest magnetically homogeneous brain region, allowing quantification of metabolites such as glutamate with minimal susceptibility-related broadening. Biologically, our previous work in the cortex [17] considered that metabolic processes like acetate uptake and glia-neuron interactions are similar across cortical regions in rats and humans. Measuring occipital metabolism should reflect broad cortical metabolic shifts during alcohol withdrawal. However, acetate and other metabolism differs in some regions, like the thalamus, with its reduced acetate consumption capacity [64]. As new techniques are developed, we expect to examine prefrontal and subcortical regions, including limbic regions.

Targeting alternate brain energetics to combat the effects of alcohol withdrawal symptoms may pose a treatment option [65]. To our knowledge, one report exists of acetate administration to decrease withdrawal symptoms, but in only one patient [66]. A more practical alternative could be ketone bodies, which enter the brain via the same MCTs as acetate, raising their possible use as therapy for AUD [67–69]. A ketogenic diet raises ketone bodies in blood for subsequent use by the brain to replace glucose partially [70]. Previous studies [17, 65] tested this idea with the introduction of a ketogenic diet to AUD patients [65], and that work showed that the diet reduced withdrawal symptoms in AUD patients relative to those on a standard diet. A recent secondary analysis from the same group [71] further supported this hypothesis, showing lower expression of craving signatures during alcohol cue exposure in the AUD patients on the ketogenic diet, compared to those on a standard American diet. The effect could be ascribed to causes, one being weaning from acetate via consumption of other monocarboxylic acid sources during recovery. Acute ketone supplementation in healthy volunteers lowered breath and blood alcohol levels and reduced subjective alcohol liking and wanting, with convergent evidence from a parallel rodent study, suggesting that ketones can modulate both the pharmacokinetics and reinforcing effects of alcohol [72]. Beyond acute energetics, ketosis has also been proposed to shift microglia toward a more anti-inflammatory phenotype and attenuate neuroinflammation in preclinical models of addiction [73], opening novel avenues to develop therapeutics for addiction. However, we acknowledge that until rigorous, double-blinded studies of acetate or ketone administration are performed, this extrapolation from our findings remains hypothetical.

### Limitations

The stratification criteria for LD and HD were based on self-reported metrics, while the AUD groups were treatment seekers, including diagnosis. Self-reported metrics through in-person interviews and the TLFB questionnaire have potential for bias [74], a long-standing challenge for objective evaluation of alcohol use. This study was designed with a selection process involving phone screening, laboratory tests including Liver Function Tests, medical chart review, in-person interview with administration of the SCID, and physical examination. Future work would benefit from the inclusion of an objective measure such as PEth [75]. The prevalence of smoking varied among the groups could potentially affect the study outcomes, but as was the case for sex effects, cell sizes were too small to address smoking’s impact. One potential impact, despite the washout period of ≥48 h, was benzodiazepine use during withdrawal, but that can only be avoided by recruiting people with mild enough AUD to forego benzodiazepines. A potential limitation is that acetate was administered intravenously, whereas in naturalistic drinking, acetate is produced hepatically: the limitation is inherent, because administering ^13^C-ethanol confounds the Glu4 and Gln4 labeling potentially with isotope directly from ethanol [18, 76, 77]. The data align with prior demonstrations that the brain shifts toward acetate oxidation during alcohol intake [16, 17, 19], particularly in heavy drinkers, suggesting that the metabolic pathways probed here are indeed physiologically engaged during real-world drinking. Due to the small number of AUD_Tx_(1W) (*n* = 9) and AUD_LTR_ (*n* = 6) subjects and the modest but still small number of LD (*n* = 13) and HD (*n* = 15) subjects, our statistical power was limited, and it was not possible to make meaningful assessments of potential factors like benzodiazepine use, smoking, and sex. Specifically, for the LD versus HD comparison, we were limited to detecting only large effects (d = 1.1) with 80% power using a two-sided test with alpha = 0.05, limited to detecting an even greater effect (d = 1.6) for the comparison of AUD_Tx_(1W) versus AUD_LTR_.

In conclusion, we observed differences in brain acetate metabolism depending on alcohol exposure. Contrary to our expectations of dramatically elevated acetate consumption in early recovery, we saw the opposite. We also expected to observe altered EPC, due to hyperexcitability in early withdrawal, but EPC appeared normal at all time points. Perhaps results would be different before detoxification or earlier in withdrawal. Maybe reduced acetate consumption is a sign of astrocyte dysregulation that reverses with longer recovery. Possibly, chronic intense drinking leads to decreased acetate transport, protecting intracerebral processes from constant acetate exposure, and then during withdrawal, the decreased availability of monocarboxylic acids contributes to symptoms of withdrawal, which supports ideas of metabolic therapy.

## Supplementary information

Supplementary Material

## Data Availability

The data were planned and acquired before data sharing agreements were common, and consent for sharing was not obtained. Therefore, the Yale IRB has said that this study’s data cannot be shared.
