# Supplementary material for: Reversible alterations of brain acetate metabolism associated with alcohol consumption
Source: Neuropsychopharmacology. 2026 May 30;51(10):1758–66. doi: 10.1038/s41386-026-02455-6 (PMC13334440; doi:10.1038/s41386-026-02455-6)
Supplement: Supplementary file 1 [file 41386_2026_2455_MOESM1_ESM.pdf]

Supplementary Material

	HD	LD	AUD_{Tx}(1W)	AUD_{Tx}(1M)	AUD_{LTR}
N	15	15	14	11	6
Age (yr)	27±8	25±4	38±8	38±8	33±6
Sex	F7, M8	F6, M9	F2, M12	F2, M9	F1, M5
Race/Ethnicity*	A5, B2, L1, W7	A3, B4, L1, W7	B8, NA1, W5	B5, NA1, W5	B1, NA1, W4
Education scale (SCID)	6±1	6±2	3.6±1.3	3.6±1.3	4±1
BMI	24.5±3.9	24±2	25.9±3.8	25.9±3.8	28±3
AST (U/L)	26±14	22.5±11	32±14	29±6.5	22±13
ALT (U/L)	23±19	17±9	30±10	30±8	21±9
Bilirubin (mg/dL)	0.6±0.3	0.52±0.25	0.5±0.2	0.44±0.13	0.63±0.23
B12 (140-960) (pg/mL)	394±103	513±146	489±168	473±192	576±8
Folate (ng/mL)	18±6	15.7±2.9	19±7	10.6±5.3	19±7
History Etoh abuse	N=4	N=0	N=14	N=11	N=
Family history of Etoh abuse	N=4	N=0	N=3	N=3	N=3
Age hit abuse (yr)	22±6	No	18±5	18±3	16-23
Age start drinking (yr)	17±2	20±3	15±2	15±1	14.3±0.8
Age of heaviest drinking (yr)	23±4	21±3	33±10	41±8	25±5
Age hit dependence (yr)	n.a.	n.a.	22±4	23±6	20±3
Months of abuse	0	n.a.	55±42	38±26	36±25
Smokers	N=3	N=0	N=10	N=8	N=4
Intake Fagerström	1.3±1.8	0	4±3	55±2.7	6±2.3
Days since last drink	2.2±0.6 (2-4)	54±80 (2-250)	4-11	29±7 (21-37)	1684±1825 (182-5000)
Drinks past 30 days	76±29	2±2	389±184	0	0
Drinks past 60 days	156±51	4±4	790±363	389±184	0
Benzo required for detox	n.a.	n.a.	N=10	N=6	n.a.
Blood-glucose before/after infusion (mg/dL)	95±9/103±18	90±15/99±28	100±15/108±22	103±15/108±14	95±12/98±15
CIWA at scan	0 except one person at 1	0	0 except one person at 2	0	0

Table S1. Demographic data for acetate infusion study (mean±SD, (min-max)). HD and LD groups had similar levels of education, whereas the AUD_{Tx}(1W) and AUD_{LTR} groups scored lower. One person in the HD group, one person in the AUD_{LTR} group and 2 people in AUD group had BMI >30, with all other participants falling within the normal BMI range. Liver function tests

AST, ALT, bilirubin, B_{12}, and folate showed no significant abnormalities in all participants. The mean age of the first drink for the AUD_{Tx}(1W) and AUD_{LTR} groups was 15 years and 14 years, respectively, lower than the HD and LD groups' 17 and 20 years, respectively. History of Etoh abuse is based on DSM-IV criteria, with four of the HD group meeting for abuse but none for dependence. The average number of drinks consumed in the past 60 days of the AUD_{Tx}(1W) group was 5-fold higher than in the HD group. *For Race/Ethnicity, A = Asian, B = Black, L = Latin, NA=Native American, W = White.

Supplement Material – Detailed Inclusion and Exclusion Criteria

Subjects were excluded from the study if they presented with bipolar disorder or schizophrenia, a history of serious medical or neurological illness that could complicate the diagnosis, psychoactive substance dependence (and possible abuse) within the preceding 6 months, mental disorders due to general medical conditions, or implanted metallic devices. Subjects were excluded if they manifested signs of major medical or neurological illness on examination or in the results of their laboratory studies. Care was taken to exclude women who were pregnant, because pregnancy may affect insulin resistance and glucose levels, thereby potentially altering monocarboxylic acid transporters (MCT) and utilization; furthermore, it would have been unethical to expose the fetus to unknown risks of acetate infusions. Women were instructed either to abstain from sexual intercourse or to use a reliable form of birth control while participating in this project, and they underwent a pregnancy test before receiving any infusions in the study. Likewise, women who were breast-feeding were not studied, because hormonal conditions at that time have potential to alter the results and we did not want to pass elevated acetate to the nursing baby.

Inclusion Criteria:

a. Aged 21-55 years and able to give voluntary written informed consent. The upper age of 55 was chosen to reduce the potential of age-related changes in brain metabolism [77].

b. HD and AUD_{Tx}(1W) must have been drinking heavily for at least 1 year.

c. For HD, consume alcohol at a typical rate of above a level of 14 drinks per week for men and consume at least 4 drinks per day at least once per week (for men). For women, consume alcohol in excess of 11 drinks per week and exceed 3 drinks per day at least once per week. Note that these criteria are somewhat lower than those used by NIAAA (2006). HD had to be non-treatment seeking.

d. AUD_{Tx}(1W) individuals had to meet SCID-I criteria for current alcohol dependence and must be willing to stay in an in-patient setting for up to 30 days.

e. For LD, over the past year typically consume less than 3 drinks per week and no more than two drinks on any given day.

f. For AUD_{LTR}, consume no alcohol in previous 6 months.

Exclusion Criteria:

a. Any medical condition or medication that, in the opinion of the study investigators, will interfere with the safe completion of the study; head trauma resulting in sustained loss of consciousness (to be evaluated on a case-by-case basis by study physician), CVA, or neurodegenerative disorder.

b. Clinical evidence for significant liver disease, such as ascites or hepatosplenomegaly.

c. For HD and LD, inability to abstain from alcohol for 24 hours, to reduce risks and to avoid confounding results with alcohol withdrawal.

d. Endocrine disorders, including diabetes, hypothyroidism.

e. For HD and LD, history of ethanol dependence or detoxification with medications or withdrawal symptoms scoring 3 or higher on the CIWA.

f. For all groups, diagnosis of current psychiatric disorder that is deemed not stable by the study physician. Exceptions:

1) Diagnosis of nicotine dependence within the past 6 months

2) AUD subjects may have a diagnosis of alcohol dependence

3) HD may have a diagnosis of alcohol abuse but not dependence.

g. For all groups, lifetime history of bipolar disorder or schizophrenia.

h. Pregnant or lactating women.

i. Clinically significant laboratory abnormalities, including but not limited to, LFTs > 4x the upper limit of normal reference range, hematocrit < 37% (males) or <33% (females), hypokalemia (potassium <3.3), serum creatinine > 1.5mg/dl, T4 ≥ 4.

j. Use of any medication that would interfere with study assessments, including anticonvulsants. Use of medications provided during the medical detoxification will be reviewed by a study physician on a case-by-case basis.

k. Implanted metallic devices or objects that could potentially prove harmful when exposed to MRS.
